# Optimal Secretory Expression of Acetaldehyde Dehydrogenase from *Issatchenkia terricola* in *Bacillus subtilis* through a Combined Strategy

**DOI:** 10.3390/molecules27030747

**Published:** 2022-01-24

**Authors:** Jing Lu, Yu Zhao, Yu Cheng, Rong Hu, Yaowei Fang, MingSheng Lyu, Shujun Wang, Zhaoxin Lu

**Affiliations:** 1 Jiangsu Key Laboratory of Marine Bioresources and Environment/Jiangsu Key Laboratory of Marine Biotechnology, Jiangsu Ocean University, Lianyungang 222005, China; jinglu@jou.edu.cn (J.L.); zy1062303721@163.com (Y.Z.); c18261380272@163.com (Y.C.); 14762391755@163.com (R.H.); foroei@163.com (Y.F.); mslyu@jou.edu.cn (M.L.); sjwang@jou.edu.cn (S.W.); 2 College of Food Science and Technology, Nanjing Agricultural University, Nanjing 210095, China; 3 Co-Innovation Center of Jiangsu Marine Bio-Industry Technology, Jiangsu Ocean University, Lianyungang 222005, China; 4 College of Food Science and Engineering, Jiangsu Ocean University, Lianyungang 222005, China; 5 Jiangsu Marine Resources Development Research Institute, Jiangsu Ocean University, Lianyungang 222000, China

**Keywords:** acetaldehyde dehydrogenase, *Bacillus subtilis*, signal peptide, tandem promoter, optimization of fermentation

## Abstract

Acetaldehyde dehydrogenases are potential enzyme preparations that can be used to detoxify acetaldehyde and other exogenous aldehydes from pharmaceuticals, food, and biofuel production. In this study, we enhanced the expression of acetaldehyde dehydrogenase sourced from *Issatchenkia terricola* (istALDH) in *Bacillus subtilis* using a combinatorial strategy for the optimization of signal peptides, promoters, and growth conditions. First, a library of various signal peptides was constructed to identify the optimal signal peptides for efficient istALDH secretion. The signal peptide yqzG achieved the highest extracellular istALDH activity (204.85 ± 3.31 U/mL). Second, the aprE promoter was replaced by a constitutive promoter (i.e., P43) and an inducible promoter (i.e., Pglv), resulting in 12.40% and 19.97% enhanced istALDH, respectively. Furthermore, the tandem promoter P43-Pglv provided a better performance, resulting in 30.96% enhanced istALDH activity. Third, the production of istALDH was optimized by testing one factor at a time. Physical parameters were optimized including the inducer (e.g., maltose) concentrations, incubation temperatures, and inoculation amounts, and the results were 2.0%, 35 °C, and 2.0%, respectively. The optimized medium results were 2.0% glucose, 1.5% peptone, 2.5% yeast extract, 1% NaCl, and 0.5% (NH_4_)_2_SO_4_. The extracellular istALDH activity was 331.19 ± 4.19 U/mL, yielding the highest production reported in the literature to date.

## 1. Introduction

Aldehyde dehydrogenases (ALDHs) can catalyze the oxidation of endogenous and exogenous aldehydes to their corresponding carboxylic acids [1]. The oxidation is coupled with a reduction in either NAD+ or NADP+, with some ALDHs displaying dual-coenzyme specificity [2]. Aldehydes (e.g., acetaldehyde (AA)) are widely present in nature, and most are toxic, mutagenic, or carcinogenic [3,4,5]. AA can be found in food, tobacco smoke, beverages and industrial products [6]. ALDHs can oxidize acetaldehyde to acetic acid, which is an effective way to eliminate acetaldehyde [7]. However, most natural ALDHs are extracted from animal liver, pancreas, or microorganisms, making it difficult to achieve large-scale production. Gene cloning, heterologous expression, and microbial fermentation of the enzyme are the best ways to obtain purified ALDHs.

In previous work, *Issatchenkia terricola* acetaldehyde dehydrogenase (istALDH) has been expressed in *Escherichia coli* with a productivity of 442.3 U/mL, which have higher ALDH activity, as compared to other ALDHs [6]. It is well known that *E. coli* strains contain endotoxins that may cause pyrogenic and shock reactions in mammals, including human [8]; therefore, they are not suitable for producing enzymes for use in the food industry. The safety of bioengineered microorganisms and their transformed products in the food industry has attracted more and more public attention [9]. Therefore, developing a host strain that is food grade and stable is essential. *Bacillus subtilis* is well known as a “generally regarded as safe”(GRAS) strain in the food industry and can be used as a microbial factory for large-scale modern industrial fermentation and production [10]. *B. subtilis* also has advantages, such as the absence of significant codon bias, non-pathogenicity, and high amenability for genetic engineering [11]. In addition, a highly efficient secretory signal peptide and molecular chaperone system result in *B. subtilis* having a superior ability to secrete homologous proteins [12]. Many high-efficiency expression systems have been constructed to achieve the expression of target proteins in *B. subtilis* [13,14]. However, attempts to produce foreign proteins using *Bacillus* strains have failed or led to disappointing yields [15], and the expression of ALDHs in *B. subtilis* has not yet been reported.

Expression components, such as signal peptides (SPs) and promoters optimized for homologous proteins, can improve their expression levels. To enhance the secretory production of recombinant proteins, strong promoters and efficient SPs are essential. It is particularly important to use a high-throughput platform to identify expression elements suitable for the target protein expression from a large number of samples in order to find the optimal SPs and promoters for the expression of specific heterologous proteins [16]. Feng et al. increased the extracellular production of L-asparaginase (ASN) in *B. subtilis* by SP screening, promoter mutation, and N-terminal deletion [17]. Employing the screening of SPs, the N-terminal structure and substitution, and the gold and silver knockout, Su et al. increased the yield of the target protein (i.e., α/β-cyclodextrin glycosyltransferase) in *B. subtilis,* which was 2.3 times higher than the control [18]. Guan et al. constructed a secretory expression system with a dual promoter (i.e., P_gsiB_-P_HpaII_) and an efficient SP (i.e., yncM) with an extracellular aminopeptidase (AP) activity of 88.86 U/mL [19].

In this study, we constructed a panel of istALDH expression conditions in *B. subtilis* that screened for various SPs to find which SP could offer an optimal secretion of istALDH. To further investigate the expression level, the promoter, the physical parameters, and the medium components were investigated and optimized. By directing the tandem promoter P_43_-P_glv_, the yqzG signal peptide, and the optimized fermentation conditions, a high-level of istALDH was achieved in a shake flask (331.19 ± 4.19 U/mL), which is the highest ALDH production level reported in the *B. subtilis* expression system, to date.

## 2. Materials and Methods

### 2.1. Strains and Plasmids

The bacterial strains and plasmids used in this study are shown in Table 1. *E. coli* DH5α was used for cloning work. *B. subtilis* strain RIK1285 and the pBE plasmid (Takara Bio, Dalian, China) were used as the host to express istALDH. The pET32a/istALDH plasmid vector [6] was used to clone the istALDH gene. The pHCMC04-pP_glv_ plasmid vector [20] was used to clone the P_glv_ promoter. The pPSQ plasmid vector [21] was used to clone the P_43_ promoter. All the chemical reagents were purchased from Solarbio (Beijing, China).

### 2.2. Media and Growth Conditions

The bacterial strains were grown in LB medium at 37 °C under agitation at 180 rpm. When appropriate, *B. subtilis* growth medium was supplemented with kanamycin (kan) at 10 μg/mL, and *E. coli* growth medium was supplemented with ampicillin (amp) at 100 μg/mL. *B. subtilis* strains were grown at 37 °C under agitation at 200 rpm to produce istALDH in the medium containing 10 g/L glucose, 15 g/L peptone, 25 g/L yeast extract, 10 g/L NaCl, and 20 g/L (NH_4_)_2_SO_4_ (pH 7.0) [20]. The volume of seed culture was 30 mL in 100 mL Erlenmeyer flask, and the volume of fermentation culture was 50 mL in 250 mL Erlenmeyer flask.

### 2.3. Construction of a Plasmid Library of istALDH Fused with Different SPs

The *istALDH* gene was amplified using primer pairs P1 and P2 (Table 2). The amplified DNA was inserted into the *Sac* I–*BamH* I site of the pBE to yield the recombinant plasmid pBE/istALDH.

The linearized-expression plasmid was prepared by PCR amplification using primer pairs P3 and P4 (Table 2) to replace the SP aprE randomly with one of the 173 different SPs by using *B. subtilis* Secretory Protein Expression System Kit (Takara Bio, Dalian, China), which thereby yielded a mixture of recombinant plasmids. The plasmid mixture was transformed into *E. coli* DH5α. The colonies on the plates were then collected and mixed, and an aliquot of the mixture was used to inoculate LB-medium-containing ampicillin to prepare a recombinant plasmid library.

*B. subtilis* RIK1285 was transformed with the plasmid library, and approximately 2500 transformants were examined in 96-deep-well plates. After 36 h of incubation at 37 °C under agitation at 250 rpm, the extracellular activity of istALDH was determined, as described previously, at OD_580_ [22] by using μQuant™ Microplate Spectrophotometer (BioTek Instruments, Inc., New York, NY, USA). Each 200 μL reaction mixture contained 20 μL supernatant, 30 μL dilution buffer (50 mmol/L pH 9.0 Tris–HCl buffer), and 150 μL of reagent solution (dilution buffer contained 300 μmol/L NAD^+^, 300 μmol/L acetaldehyde, 300 μmol/L nitroblue tetrazolium, and 30 μmol/L phenazine methosulfate). Finally, the suspected positive clones were re-tested as above in shake flasks and then confirmed positive clones that could obtain higher istALDH activity were selected for further sequencing analysis.

### 2.4. Construction of Plasmids Expressing the istALDH with Different Promoter

First, the gene of Pglv was inserted into the pBE/istALDH-1 to yield the recombinant plasmid pPglv/istALDH by using an In-Fusion HD Cloning Kit (Takara Bio, Dalian, China) and primer pairs P5–P8 (Table 2). Second, by using primer pairs P7–P10 (Table 2), the promoter Pglv was replaced by P_43_, yielding the recombinant plasmid pP_43_/istALDH. Third, primer pairs P7 and P11–P16 (Table 2) were used for the recombinant plasmids pPglv-P43/ist-ALDH and pP43-Pglv/ist-ALDH. After sequence verification, all the recombinant plasmids were used to independently transform *B. subtilis* RIK1285.

### 2.5. Assay and Detection of Secreted istALDH

The activity of istALDH was determined, as described previously with minor modifications, using a UV-2450 spectrophotometer (Shimadzu UV-2550, Tokyo, Japan) at 40 °C [23]. The 3 mL reaction mixture contained 2000 μL 50 mmol/L Tris–HCl buffer (pH 9.0), 100 μL 1% acetaldehyde, 400 μL 2% NAD^+^, 400 μL 0.75 mol/L KCl, and 100 μL enzymatic extract. One unit was defined as the amount of enzyme producing 1 μmol NADH per minute. The activity of extracellular istALDH was determined after centrifugation, and the activity of intracellular istALDH was determined after the strain was broken via ultrasound.

### 2.6. Quantitative Real-Time PCR Analysis of istALDH Expression in Recombinant B. subtilis

Total RNA was isolated using TransZol Up and reverse-transcribed into cDNA with the Hi Script II 1st Strand cDNA Synthesis Kit (+g DNA wiper) (Vazyme Biotech Co., Nanjing, China). Quantitative real-time PCR analysis was performed, as described previously [24], with ABI Step One Plus sequence detection system (Applied Biosystems, Foster City, CA, USA) by using HieffTM qPCR SYBR^®^ Green Master Mix (Yeasen, Shanghai, China). The 2^−ΔΔCt^ method was used to calculate the relative expression lever of *istALDH* (primers were listed in Table 2).

## 3. Results and Discussion

### 3.1. Heterologous Production of Secreted istALDH in B. subtilis Strain RIK1285

**Figure 1:**
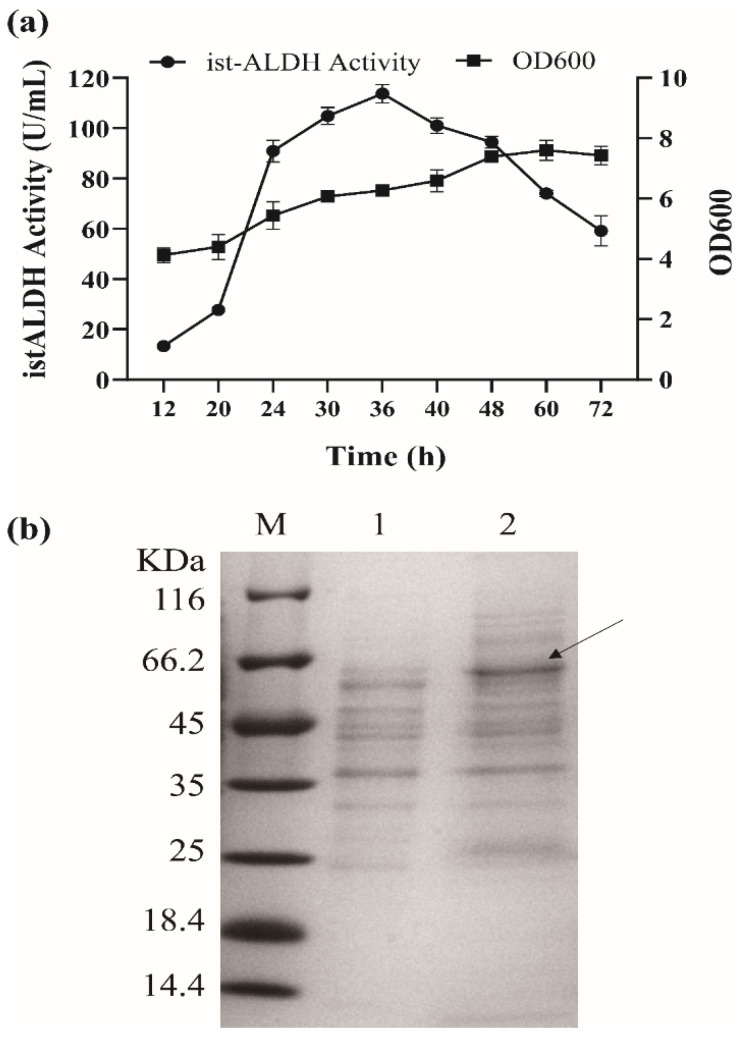
(**a**) The recombinant istALDH activity and growth curve of *B. subtilis* RIK 1285 harboring pBE/istALDH. (**b**) SDS-PAGE analysis of expression products for the recombinant istALDH at the fermentation supernatant expected molecular mass of 57 k Da (indicated by the black arrow). Lane M is the molecular mass marker (26610, Thermo). Lane 1 (negative control) is the fermentation supernatant of RIK 1285 harboring pBE. Lanes 2 is the fermentation supernatant of RIK 1285 harboring pBE/istALDH. The experimental data were expressed as means ± standard deviations (SDs) of at least three independent experiments.

The istALDH was a highly active enzyme and has the potential to be used for acetaldehyde detoxification in the food industry. The enzyme has been expressed in *E. coli* [6]. In this study, the *istALDH* gene was first cloned to construct the recombinant plasmid pBE/istALDH, which was expressed under the control of the aprE promoter. The extracellular istALDH activity in BS-1 was 113.71 ± 3.62 U/mL at 36 h (Figure 1a). The heterologous expression of istALDH in the *B. subtilis* strain RIK1285 was confirmed by SDS-PAGE, which showed an extra band of the estimated protein size of 57 k Da in the culture extract (Figure 1b). To the best of our knowledge, this may be the first time the heterologous secretory expression of istALDH in *B. subtilis* has been reported.

### 3.2. Effect of the Signal Peptides on the Secretion of istALDH

As described above, the aprE SP was replaced with different SPs in an optimization experiment. More than 2000 independent colonies were inoculated randomly into separate wells of a 96-well plate. The sequencing analysis of the SPs resulting from the pBE/istALDH derivatives enabled the determination of additional, varied SPs displaying a higher efficiency of secretion, as compared to the SP aprE (Table 3). The SP yqzG enhanced the secretion of the istALDH efficiently, and the extracellular activity was 204.85 ± 3.31 U/mL in a shake flask for 36 h, which was a 54.53% increase, as compared to using the aprE SP. The protein yqzG is a putative exported protein of *B. subtilis*, and to the best of our knowledge, this may be the first reported use of SP yqzG used for the secretory expression of heterologous proteins.

In previous reports, the charge distribution of the N-domain and the hydrophobic H-domain of signal peptides could affect the secretion [25,26,27]. In the protein translocation process, the positive charge (or basic residues) may play several roles, enabling the SP to insert itself into the inner membrane by forming a loop with the negatively charged lipid head groups through electrostatic interactions [28]. In *B. subtilis*, hydrophobicity is a key determinant for SP to bind to the Ffh component of the signal recognition particle (SRP) or to the trigger factor, and SRP can discriminate between signal peptides with relatively high hydrophilicities [25]. The SignalP 4.1 server has been widely used to predict signal peptides, and the D-score has been used to discriminate signal peptides from non-signal peptides; however, the scores do not necessarily correlate with secretion efficiency [29]. According to the results shown in Table 3, the higher efficiency SPs appeared to have either a higher D-score, charge, or hydrophilicity.

However, the predicted SP with the highest secretion efficiency was xynA with a D-score of 0.757, whereas experimentally, the SP yqzG (D-score = 0.716) showed the best secretion efficiency for istALDH, with the same result also found in charge and hydrophobicity. Therefore, the properties of the signal peptides may have affected the secretion efficiency, but there was no clear quantitative relationship between them. By exploring the amino acid composition of many predicted exported proteins and cytoplasmic proteins, Wang et al. discovered a preference for disorder-promoting amino acids in the N-terminal regions of mature domains, which shed light on the contributions of mature domains in membrane targeting [30]. Moreover, our screening results indicated that for istALDH, the SPs from uncommon proteins had better results than that of typical SPs (e.g., aprE and nprE), which have been widely used for protein secretion and expression. Our results were consistent with previous reports [31]. Therefore, the properties of both signal peptides and target proteins may have a combined effect on secretion efficiency, so signal peptide screening remains an effective method for improving secretion efficiency.

### 3.3. Effect of the Promoter on Secretion of istALDH

**Figure 2:**
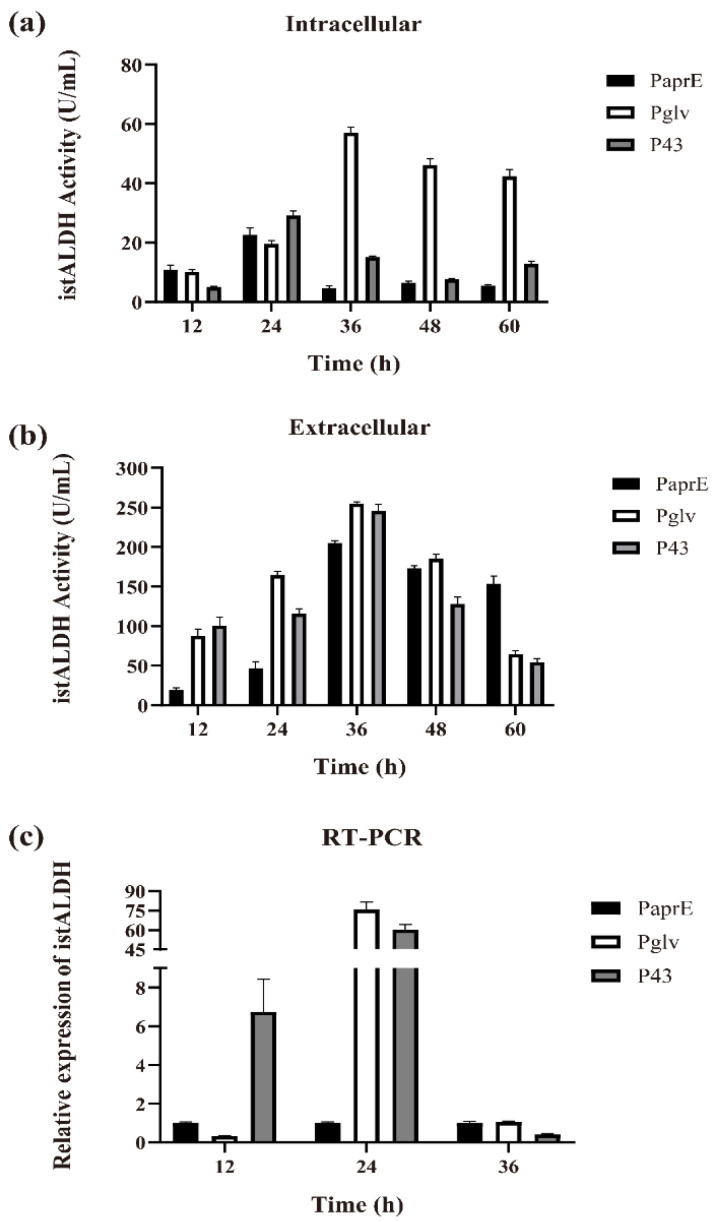
The intracellular (**a**) and extracellular (**b**) istALDH activities, and the relative expression of istALDH (**c**) of recombinant *B. subtilis* strains with different promoters. The experimental data were expressed as means ± standard deviations (SDs) of at least three independent experiments.

A controllable and efficient expression system is desirable for large-scale industrial production. Yang et al. developed a high-level expression system directed by promoter P_glv_, which was induced by maltose [32,33]. As compared to isopropyl β-d-1-thiogalactopyranoside (IPTG) and xylose, inducers for two widely used induction systems P_spac_ and P_xyl_, respectively, maltose is a cheap and safe inducer for industrial application. The promoter P_43_ is a strong, well-known constitutive promoter, and the strength of P_aprE_ is 5–10% of that of P_43_ in β-galactosidase expression [34]. Therefore, we replaced P_aprE_ with an inducible promoter (i.e., P_glv_ promoter) and a constitutive promoter (i.e., P_43_ promoter). As shown in Figure 2a,b, both the intracellular and extracellular istALDH activities in the recombinant strains reached their maximum levels at 36 h of shake-flask fermentation, resulting in the intracellular secretion of istALDH and obtained 57.00 ± 2.04 and 15.11 ± 0.42 U/mL, respectively (1123.17% and 224.25% enhanced, respectively, as compared to the P_aprE_), and the extracellular secretion of istALDH obtained 254.82 ± 2.07 and 245.76 ± 8.72 U/mL (19.97% and 12.40 % enhanced, respectively, as compared to the P_aprE_), respectively.

To further study the influence of the different promoters on the expression efficiency of *istALDH*, we evaluated the relative expression of the *istALDH* gene by RT-PCR (Figure 2c). The results showed that both P_glv_ and P_43_ could increase the expression of the *istALDH* gene after 12–24 h of fermentation, resulting in a total activity of 311.82 ± 3.01 and 260.87 ± 5.72 U/mL, respectively (Figure 2a,b). However, the increase in gene expression was much greater than the increase in istALDH production. A possible reason may be that the speed of the gene transcription was faster than that of protein translation while the transcribed mRNA was too late to be translated into the target protein at such a speed.

**Figure 3:**
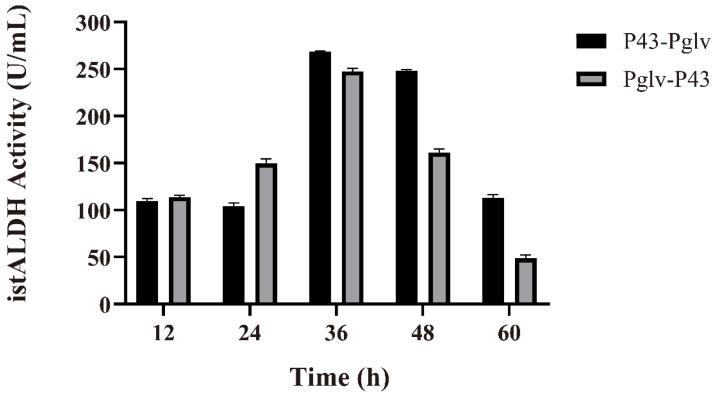
The istALDH activity of recombinant *B. subtilis* strains with tandem promoters. The experimental data were expressed as means ± standard deviations (SDs) of at least three independent experiments.

Many studies have shown that two or more tandem promoters can significantly improve the expression levels of heterogeneous genes. The tandem promoters fused α-amylase and maltogenic amylase promoter downstream of P_HpaII_, respectively. The TSαGT productivity was 11-fold and 12-fold higher than that of P_HpaII_ [35]. To explore the possibility of tandem promoters enhancing the secretion of istALDH, the promoters P_43_-P_glv_ were constructed. As a result, the secretion of istALDH obtained by P_43_-P_glv_ and P_glv_-P_43_ promoters were 268.26 ± 1.19 and 247.76 ± 2.90 U/mL at 36 h, respectively (Figure 3).

Although the recombinant strain harbors the plasmid containing the promoters P_43_-P_glv_ showed the highest istALDH activity, the differences in expression strength between the dual-promoter and single-promoter systems were relatively weak. Zhang et al. constructed and screened nine plasmids equipped with single promoters and six plasmids equipped with dual promoters to enhance β-CGTase secretion, and the dual-promoter P_HpaII_-P_amyQ_ system was found to support the target protein secretion [36]. The strength of the promoter adjacent to the target gene had a greater impact on the transcription efficiency of the dual promoter. Therefore, we constructed a series of single dual-promoters to screen for highly efficient promoters and then used the efficient promoters to construct dual promoters to further increase the istALDH secretion yield.

### 3.4. Effect on the Fermentation Conditions on the Secretion of istALDH

**Figure 4:**
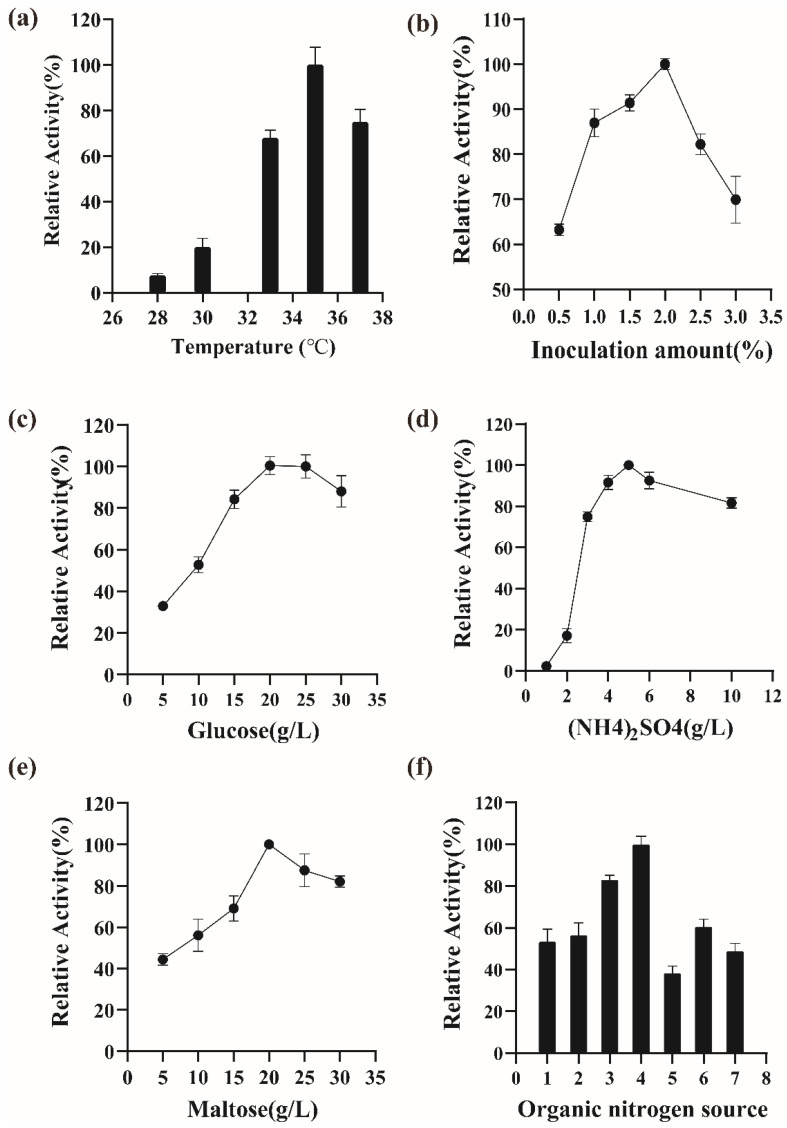
Optimization of culture conditions and medium composition of *B. subtilis*-5. (**a**) Temperature, (**b**) inoculum amount, (**c**) glucose, (**d**) NH_4_)_2_SO_4_, (**e**) maltose, (**f**) organic nitrogen source. The experimental data were expressed as means ± standard deviations (SDs) of at least three independent experiments.

The optimization of the physical parameters and the medium for the expression of istALDH were as follows: The carbon-sourced (i.e., glucose) concentrations tested were 5, 10, 15, 20, 25, and 30 g/L. The organic nitrogen-sourced media tested were various combinations of peptone and yeast extract (Table 4). The (NH_4_)_2_SO_4_ concentrations tested were 1, 2, 3, 4, 5, 6, and 10 g/L. The inducer (i.e., maltose) concentrations tested were 5, 10, 15, 20, 25, and 30 g/L. The fermentation temperatures tested were 28 °C, 30 °C, 33 °C, 35 °C, and 37 °C. The inoculation amounts tested were 0.5%, 1.0%, 1.5%, 2.0%, 2.5%, and 3.0% (*v*/*v*). According to the results (Figure 4), the best growth conditions were 15 g/L peptone, 25 g/L yeast extract, 20 g/L glucose, 20 g/L maltose, 5 g/L (NH_4_)_2_SO_4_, and a temperature of 35 °C with an inoculation amount of 2.0%. The recombinant strain BS-5 produced a maximum istALDH activity of 331.19 ± 4.19 U/mL. Future studies will focus on optimizing the fermentation process of BS-5 in a fermenter to further improve the istALDH yield.

## 4. Conclusions

To the best of our knowledge, this study was the first to report on the optimization of the secretion for istALDH in *B. subtilis* via SP screening and promoter replacement. The SP ypzG and tandem promoter P_43_-P_glv_ were the best candidates for istALDH secretion. The recombinant strain BS-5 produced a maximum istALDH activity of 331.19 ± 4.19 U/mL under the following growth conditions: 15 g/L peptone, 25 g/L yeast extract, 20 g/L glucose, 20 g/L maltose, 5 g/L (NH_4_)_2_SO_4_, and a temperature of 35 °C with an inoculation amount of 2.0%, the highest level reported in a shake flask to our knowledge. This work demonstrated that optimizing signal peptides and promoters are a promising way to enhance istALDH expression in *B. subtilis*. In the future, additional studies should focus on high-cell-density cultivation and to further improve istALDH production.

## Figures and Tables

**Table 1:** Bacterial strains and plasmids used in this study.

Strains/Plasmids	Characteristics *	Source
Strains		
*E.coli* DH5α	deoR endA1 gyrA96 hsdR17 (rk-mk+)recA1 relA1 supE44 thi-1 Δ(lacZYA-argF)U169 Φ80lacZ ΔM15F-λ-	Vazyme Biotech
*B. subtilis* RIK1285	Marburg 168 derivative; trpC2, lys1, aprEΔ3, nprR2, nprE18	Takara
*B. subtilis* BS000	derived from *B. subtilis* 168, amyE::promoter-signal peptide	laboratory stock
BS-1	RIK1285 harboring pBE/istALDH	current study
BS-2	RIK1285 harboring pBE/istALDH-1	current study
BS-3	RIK1285 harboring pP_glv_/istALDH	current study
BS-4	RIK1285 harboring pP_43_/istALDH	current study
BS-5	RIK1285 harboring p P_43_-P_glv_ /ist-ALDH	current study
BS-6	RIK1285 harboring p P_glv_-P_43_/ist-ALDH	current study
Plasmid		
pMD19T	TA clon vector, Amp^+^	Takara
pHCMC04-pPglv	P_glv_ promoter	[20]
pPSQ	P_43_ promoter	[21]
pET32a/istALDH	istALDH	[6]
pBE	Kan^+^, Amp^+^, PaprE, SPaprE	Takara
pBE/istALDH	Kan^+^, Amp^+^, P_aprE_, SPaprE, istALDH	current study
pBE/istALDH-1	Kan^+^, Amp^+^, P_aprE_, SPyqzG, istALDH	current study
pPglv/istALDH	Kan^+^, Amp^+^, P_glv_, SPyqzG, istALDH	current study
pP43/istALDH	Kan^+^, Amp^+^, P_43_, SPyqzG, istALDH	current study
pP43-Pglv/istALDH	Kan^+^, Amp^+^, P_43_-P_glv_, SPyqzG, istALDH	current study
pPglv-P43/istALDH	Kan^+^, Amp^+^, P_glv_-P_43_, SPyqzG, istALDH	current study

* Amp^+^ and Kan^+^ indicate resistance to ampicillin and kanamycin, respectively.

**Table 2:** Primers used in this study.

Primer	Sequence (5′ to 3′)
P1	GAGCTCCTTAGAACTGCAACTAGAAC
P2	GGATCCTTGTGGGCCATCGTTAATGGC
P3	CGCGTCCCTCTCCTTTTGCTTAAGTTCAGAGTAG
P4	GGCCGGTGCACATATGGAGCTCCTTAGAACTGC
P5	GCCGTCTGTACGTTCCTAAACTAGTGGCATGTATCCGAATC
P6	GTTTGATCATCATACGCGTACGACCTCCTTGATAACG
P7	ATGATGATCAAACAATGTGTGATTTGTC
P8	TTAGGAACGTACAGACGGCTTAAAAG
P9	CTTTTAAGCCGTCTGTACGTTCCTAAACTAGTATATTCCTTTTGATAGGTGG
P10	GACAAATCACACATTGTTTGATCATCATACGCGTATTCCTCTCTTACCTATAATG
P11	GCCGTCTGTACGTTCCTAAACTAGTATATTCCTTTTGATAGGTGGTATG
P12	CTTTTGTACGATTCGGATACATGCCACTAGTATTCCTCTCTTACCTATA
P13	GGCATGTATCCGAATCGTACAAAAG
P14	ACTAGTTTAGGAACGTACAGACGGCTTAAAAG
P15	CTTTTAAGCCGTCTGTACGTTCCTAAACTAGTGGCATGTATCCGAATC
P16	CCACCTATCAAAAGGAATATACTAGTACGACCTCCTTGATAACG
ALDH-RT-F	CAGAGGTGAGCACTTGATGAAAC
ALDH-RT-F	CAGCCCAACCAGCACAATACTTA
16S rRNA-F	ACGGGAGGCAGCAGTAGGG
16S rRNA-R	ACGGGAGGCAGCAGTAGGG

**Table 3:** Efficiency of different SPs in istALDH secretion.

Signal Peptide	Protein Sequence	Len	D-Score ^a^	Charge ^b^	Hydrophilicity ^c^ (%)	Activity of istALDH ^d^ (U/mL)
yqzG	MMIKQCVICLSLLVFGTTAAHA	22	0.716	1	63.64	204.85 ± 3.31
wapA	MKKRKRRNFKRFIAAFLVLALMISLVPADVLA	32	0.707	7	65.63	184.81 ± 3.59
yngK	MKVCQKSIVRFLVSLIIGTFVISVPFMANA	30	0.677	3	66.67	160.76 ± 3.86
xynA	MFKFKKNFLVGLSAALMSISLFSATASA	28	0.757	1	64.29	147.84 ± 2.90
pbp	MKKSIKLYVAVLLLFVVASVPYMHQAALA	29	0.672	3	68.97	140.20 ± 3.52
aprE	MRSKKLWISLLFALTLIFTMAFSNMSVQA	29	0.644	3	62.07	113.71 ± 3.62

^a^ D-score calculated using Signal P 4.1. ^b^ Charge of the SP was calculated based on D and E counted as −1, R; K as +1; and any other amino acid as 0. ^c^ Percentage of hydrophobic amino acids in each signal sequence was calculated according to G, A, V, L, I, M, F, W, and P as hydrophobic amino acids, whereas all others were hydrophilic. ^d^ Values represent extracellular istALDH activity ± SD based on three independent results.

**Table 4:** Different combinations of peptone and yeast extract.

g/L	1	2	3	4	5	6	7
Peptone	0	10	15	20	25	30	40
Yeast extract	40	30	25	20	15	10	0

## Data Availability

The data presented in this study are available in the present article.

## References

[1] Sophos N.A., Pappa A., Ziegler T.L., Vasiliou V. (2003). Aldehyde dehydrogenase gene superfamily: The 2000 update. Chem. Biol. Interact..

[2] Gonzálezsegura L., Riverosrosas H., Juliánsánchez A., Muñozclares R.A. (2015). Residues that influence coenzyme preference in the aldehyde dehydrogenases. Chem. Biol. Interact..

[3] Dellarco V.L. (1988). A mutagenicity assessment of acetaldehyde. Mutat. Res. Rev. Genet. Toxicol..

[4] Maffei F., Fimognari C., Castelli E., Stefanini G.F., Forti G.C., Hrelia P. (2000). Increased cytogenetic damage detected by FISH analysis on micronuclei in peripheral lymphocytes from alcoholics. Mutagenesis.

[5] Lachenmeier D.W., Sohnius E.M. (2008). The role of acetaldehyde outside ethanol metabolism in the carcinogenicity of alcoholic beverages: Evidence from a large chemical survey. Food Chem. Toxicol..

[6] Yao Z., Lu F., Bie X., Lu Z. (2012). Gene cloning, expression, and characterization of a novel acetaldehyde dehydrogenase from Issatchenkia terricola strain XJ-2. Appl. Microbiol. Biotechnol..

[7] Lu J., Zhu X., Zhang C., Lu F., Lu Z., Lu Y. (2020). Co-expression of alcohol dehydrogenase and aldehyde dehydrogenase in *Bacillus subtilis* for alcohol detoxification. Food Chem. Toxicol..

[8] Schädlich L., Senger T., Kirschning C.J., Müller M., Gissmann L. (2009). Refining HPV 16 L1 purification from *E. coli*: Reducing endotoxin contaminations and their impact on immunogenicity. Vaccine.

[9] Kai H., Xiao G., Bo J., Li S. (2018). Construction of a food-grade arginase expression system and its application in L-ornithine production with whole cell biocatalyst. Process Biochem..

[10] Vavrová L., Muchová K., Barák I., Felice M.D., Fouet A., Barák I., Cutting S., Ricca E. (2010). Comparison of different *Bacillus subtilis* expression systems. Res. Microbiol..

[11] Harwood C.R., Cranenburgh R. (2008). *Bacillus* protein secretion: An unfolding story. Trends Microbiol..

[12] Wang Y., Miao Y., Hu L.-p., Kai W., Zhu R. (2020). Immunization of mice against alpha, beta, and epsilon toxins of *Clostridium* perfringens using recombinant rCpa-b-x expressed by *Bacillus subtilis*. Mol. Immunol..

[13] Dong H., Zhang D. (2014). Current development in genetic engineering strategies of *Bacillus* species. Microb. Cell Factories.

[14] Zhou C., Ye B., Cheng S., Zhao L., Liu Y., Jiang J., Yan X. (2019). Promoter engineering enables overproduction of foreign proteins from a single copy expression cassette in *Bacillus subtilis*. Microb. Cell Factories.

[15] Mao S., Zhang Z., Ma X., Tian H., Liu Y. (2021). Efficient secretion expression of phospholipase D in Bacillus subtilis and its application in synthesis of phosphatidylserine by enzyme immobilization. Int. J. Biol. Macromol..

[16] Peng C., Shi C., Cao X., Li Y., Liu F., Lu F. (2019). Factors influencing recombinant protein secretion efficiency in Gram-positive bacteria: Signal peptide and beyond. Front. Bioeng. Biotechnol..

[17] Feng Y., Liu S., Jiao Y., Gao H., Wang M., Du G., Chen J. (2017). Enhanced extracellular production of L-asparaginase from *Bacillus subtilis* 168 by *B. subtilis* WB600 through a combined strategy. Appl. Microbiol. Biotechnol..

[18] Su L., Li Y., Wu J. (2021). Efficient secretory expression of Bacillus stearothermophilus α/β-cyclodextrin glycosyltransferase in *Bacillus subtilis*. J. Biotechnol..

[19] Guan C., Cui W., Cheng J., Rui L., Liu Z., Li Z., Zhou Z. (2016). Construction of a highly active secretory expression system via an engineered dual promoter and a highly efficient signal peptide in *Bacillus subtilis*. New Biotechnol..

[20] Liu M., Xie M., Yang Y., Liu T., Liqin D.U., Liang Z., Wei Y. (2016). High Level Secretion Expression of Maltogenicα-amylase from Saccharomonospora viridis in Bacillus subtilis. Guangxi Sci..

[21] Gao L., Han J., Liu H., Qu X., Lu Z., Bie X. (2017). Plipastatin and surfactin coproduction by *Bacillus subtilis* pB2-L and their effects on microorganisms. Antonie Van Leeuwenhoek.

[22] Mayer K.M., Arnold F.H. (2002). A colorimetric assay to quantify dehydrogenase activity in crude cell lysates. J. Biomol. Screen..

[23] Bostian K.A., Betts G.F. (1978). Kinetics and reaction mechanism of potassium-activated aldehyde dehydrogenase from *Saccharomyces cerevisiae*. Biochem. J..

[24] Jing L., Yunbin L., Mingtong L., Jing S., Zhenghua H., Fengxia L., Zhaoxin L. (2018). Alleviating acute alcoholic liver injury in mice with Bacillus subtilis co-expressing alcohol dehydrogenase and acetaldehyde dehydrogenase. J. Funct. Foods.

[25] Zanen G., Houben E.N.G., Meima R., Tjalsma H., Jongbloed J.D., Westers H., Oudega B., Luirink J., van Dijl J.M., Quax W.J. (2005). Signal peptide hydrophobicity is critical for early stages in protein export by *Bacillus subtilis*. FEBS J..

[26] Ismail N.F., Hamdan S., Mahadi N.M., Ama M., Rabu A., Fda B., Klappa P., Illias R.M. (2011). A mutant l-asparaginase II signal peptide improves the secretion of recombinant cyclodextrin glucanotransferase and the viability of *Escherichia coli*. Biotechnol. Lett..

[27] Caspers M., Brockmeier U., Degering C., Eggert T., Freudl R. (2010). Improvement of Sec-dependent secretion of a heterologous model protein in *Bacillus subtilis* by saturation mutagenesis of the N-domain of the AmyE signal peptide. Appl. Microbiol. Biotechnol..

[28] Inouye M., Halegoua S., Beckwith J. (1980). Secretion and membrane Localization of Proteins in *Escherichia Coli*. CRC Crit. Rev. Biochem..

[29] Nielsen H. (2017). Predicting Secretory Proteins with SignalP. Methods Mol. Biol..

[30] Wang G., Dong Y., Chen H., Zhang H., Song Y., Chen W. (2012). High incidence of disorder-promoting amino acids in the amino terminus of mature proteins in *Bacillus subtilis*. Am. J. Biochem. Biotechnol..

[31] Miao H., Jiang R., Han N., Ma Y., Wu Q., Mu Y., Huang Z. (2021). Enhanced extracellular expression of α-Amylase DL3-4-1 in *Bacillus subtilis* via systematic screening of optimal signal peptides. Process Biochem..

[32] Ming-Ming Y., Wei-Wei Z., Xi-Feng Z., Pei-Lin C. (2006). Construction and characterization of a novel maltose inducible expression vector in *Bacillus subtilis*. Biotechnol. Lett..

[33] Yang M.M., Zhang W.W., Chen Y.L., Gong Y.S. (2010). Development of a *Bacillus subtilis* expression system using the improved P glv promoter. Microb. Cell Factories.

[34] Kim J.H., Hwang B.Y., Roh J.W., Lee J.K., Kim K., Wong S.L., Yun H.D., Lee S.G., Kim B.G. (2008). Camparison of PaprE, PamyE, and P43 promoter strength for β-galactosidase and staphylokinase expression in *Bacillus subtilis*. Biotechnol. Bioprocess Eng..

[35] Kang H.K., Jang J.H., Shim J.H., Park J.T., Kim Y.W., Park K.H. (2010). Efficient constitutive expression of thermostable 4-α-glucanotransferase in *Bacillus subtilis* using dual promoters. World J. Microbiol. Biotechnol..

[36] Kang Z., Su L., Duan X., Liu L., Jing W. (2017). High-level extracellular protein production in *Bacillus subtilis* using an optimized dual-promoter expression system. Microb. Cell Factories.

